# Ferroptosis in lung cancer: dual role, multi-level regulation, and new therapeutic strategies

**DOI:** 10.3389/fonc.2024.1360638

**Published:** 2024-03-07

**Authors:** Yunbin Li, Xiaosong Li, Jian Li

**Affiliations:** Department of Thoracic Surgery, The Affiliated Hospital of Guizhou Medical University, Guiyang, Guizhou, China

**Keywords:** lung cancer, ferroptosis, combination therapy, biomarkers, nanomedicine

## Abstract

Lung cancer is a highly prevalent malignant tumor worldwide, with high incidence and death rates. Recently, there has been increasing recognition of the role of ferroptosis, a unique cell death mechanism, in lung cancer. This review aims to summarize the current research progress on the relationship between ferroptosis and lung cancer. It also provides a comprehensive analysis of the regulatory processes of ferroptosis in various stages, including epigenetics, transcription, post-transcription, translation, and post-translation. Additionally, the review explores the dual nature of ferroptosis in lung cancer progression, which presents interesting therapeutic possibilities. On one hand, ferroptosis can promote the escape of immune surveillance and reduce the efficacy of treatment in the early stages of tumors. On the other hand, it can counter drug resistance, enhance radiosensitivity, and promote immunotherapy. The article also discusses various combination treatment strategies based on the mechanism of ferroptosis. Overall, this review offers a holistic perspective on the role of ferroptosis in the onset, progression, and treatment of lung cancer. It aims to contribute to future research and clinical interventions in this field.

## 1 Introduction

Lung cancer is a malignant tumor with high incidence and high mortality. Epidemiological statistics show that lung cancer is the leading cause of cancer-related death, with an incidence rate of 11.4% and a mortality rate of 18% among all malignant tumors in the world in 2020 (1). Studies indicate a grim forecast for lung cancer incidence, with many countries anticipating an increase by 2035 (2). While early-stage lung cancer is typically surgically treatable, most cases are diagnosed at intermediate or advanced stages, necessitating conservative treatments. Medication resistance remains a predominant challenge in lung cancer therapy, which most advanced Non-small cell lung cancer (NSCLC) patients develop resistance to current treatments, leading to a worsening of the condition (3). With a 5-year survival rate under 18% (4), the quest for new therapeutic targets, approaches to counteract drug resistance, and ways to improve patients’ quality and duration of life are pivotal in lung cancer research.

Introduced by Stockwell et al., “ferroptosis” represents a unique regulated cell death propelled by iron-dependent lipid peroxidation (5). Its morphology diverges from other forms of cell death characterized by membrane rupture, shrinkage of mitochondrial volume, reduction or disappearance of mitochondrial cristae, and an absence of nuclear condensation and chromatin marginalization. Ferroptosis’ biochemical characteristics are primarily reflected in three aspects. Firstly, the build-up of reactive oxygen species (ROS) is deemed a direct trigger for ferroptosis. Within mitochondria, the accumulation of ROS amplifies oxidative stress by inducing further ROS release through a positive feedback loop. In this milieu, polyunsaturated fatty acid phospholipids (PUFA-PL) are oxidized to form cytotoxic polyunsaturated fatty acid phospholipid hydroperoxides (PUFA-PL-OOH), which causes endoplasmic reticulum membrane stiffness and directly damages cell membranes, leading to cell death. Secondly, lipid peroxidation is considered the central feature of ferroptosis. During the process of ferroptosis, an unrestrained increase in lipid peroxidation can be observed (6). Lipid peroxidation denotes a process where oxidants extract unstable hydrogen atoms from the methylene bridges of polyunsaturated fatty acid (PUFA), culminating in the generation of a plethora of lipid peroxidation free radicals and hydrogen peroxide. During this process, Acyl-CoA synthetase long-chain family member 4 (ACSL4) and lysophosphatidylcholine acyltransferase 3 (LPCAT3) engage sequentially, facilitating the conversion of PUFA, ultimately resulting in the formation of phospholipids with PUFA-PL. Subsequently, catalyzed by lipoxygenase (LOX), these phospholipids undergo further oxidation to form PUFA- PL-OOH. LOX is widely regarded as a key regulator in modulating ferroptosis (7). In the end, in iron metabolism, the Fenton reaction involving Fe^2+^ and hydrogen peroxide produces vast hydroxyl free radicals, triggering intense oxidative stress and ROS generation, and consequently ferroptosis.

**Figure 1:**
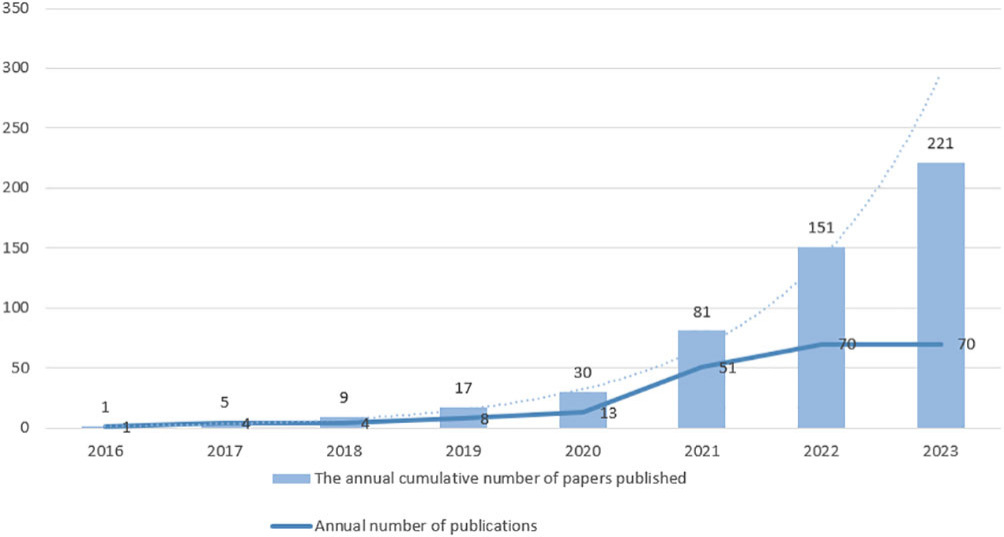
The publications on lung cancer and ferroptosis from 2016 to October 2023. The number of publications related to “lung cancer” and “ferroptosis” from 2016 to October 2023 was searched in PubMed. The broken line represents the number of annual number of publications, and the bar graph represents the annual cumulative number of papers published. The dashed line represents the predicted trend of the index, whose prediction formula is $y=0.8071e^{0.7379x}$ .

Ferroptosis may play a crucial role in regulating tumor growth. Up to now, numerous studies have been conducted on ferroptosis in relation to tumors. As a regulated cell death form, ferroptosis has been shown to stifle multiple tumors’ growth through drug and cytokine responses. Interestingly, drug-resistant cancer cells, especially those with enhanced metastatic potential, are more susceptible to ferroptosis (6). In lung cancer, especially lung adenocarcinoma, EMT occurs frequently and mainly originates from alveolar type II epithelial cells (8). EMT is associated with PD-L1 expression, especially in lung cancer, where elevated levels of PD-L1 suppress the immune system and make cancer more likely to spread (9). In addition, the integration of ferroptosis into standard treatment protocols has been investigated across a range of malignancies, including hepatocellular carcinoma, colorectal cancer, ovarian cancer, and glioblastoma (10). A surge in research is observed on ferroptosis’s influence in lung cancer, with a rise in related publications, as seen in **Figure 1**. The objective of this paper is to delve into the function of ferroptosis in lung cancer, its intricate regulatory systems, and potential biological markers, with aspirations of offering novel perspectives for lung cancer management and outcome prediction.

## 2 Ferroptosis in lung cancer

### 2.1 Ferroptosis

The primary cause of ferroptosis is the accumulation of lipid peroxides (LPO) and ROS within cells, and its mechanism can be summarized as an imbalance of the cellular antioxidant system, iron overload, and lipid peroxidation.

The cystine/glutamate antiporter system (system Xc-) situated on the cellular membrane plays a vital role in the cell’s antioxidative response. Comprising the light chain solute carrier family 7 member 11 (SLC7A11) and the heavy chain solute carrier family 3 member 2 (SLC3A2), system Xc- functions effectively. SLC7A11, a multi-pass transmembrane protein, facilitates the anti-transport activity of cystine/glutamate, exchanging extracellular cystine with intracellular glutamate at a 1:1 ratio. Once inside the cell, cystine gets reduced to cysteine and subsequently collaborates with glutamate and glycine to produce glutathione (GSH). GSH then, under the influence of glutathione peroxidase 4 (GPX4), converts LPO to cell membrane-friendly lipids, countering lipid peroxidation caused by increased ROS (11). SLC7A11 emerges as a central figure in the ferroptosis process, affected by various factors like glutamate and NF-E2-related factor 2(NRF2) ultimately determining the progression of ferroptosis. For example, NRF2 promoted SLC7A11 transcription by binding to the antioxidant response elements in the promoter region of SLC7A11 (12). Erastin treatment can reduce the occupancy of H2Bub1 in the regulatory region of SLC7A11 gene, inhibit the expression of SLC7A11, and increase the sensitivity of cells to ferroptosis (13). Moreover, specific compounds such as RAS selective lethal 3 (RSL3) and ferroptosis inducer 56 (FIN56) target the fundamental factor GPX4 to modulate ferroptosis.

In nature, iron primarily exists as Fe^2+^ and Fe^3+^. Dietary iron predominantly appears as Fe^3+^, which transforms into Fe^2+^ upon ingestion, making its way to the duodenum. Subsequently, it’s transported into intestinal epithelial cells via Divalent metal transporter 1 (DMT1) and is expelled from the basolateral membrane. It’s been observed that by modulating DMT1 expression, one can influence ferroptosis (14). Excessive intracellular Fe^2+^, when oxidized to Fe^3+^, binds to transferrin, forming a state known as the “labile pool”. Iron overload facilitates the Fenton reaction, producing a plethora of hydroxyl radicals, leading to pronounced oxidative stress and spawning a surge in ROS, a prelude to ferroptosis (15). Patel and his team found that GSH can interact with Fe^2+^ (16). A depletion of GSH not only leads to GPX4 deactivation but also propels Fe^2+^ towards the Fenton reaction, amplifying lipid peroxide production and accelerating ferroptosis (16). Hence, the regulation of iron metabolism emerges as a crucial intervention point for controlling ferroptosis.

A crucial juncture in ferroptosis is the formation of LPO. Following the involvement of acetyl-CoA in the formation of unbound PUFA, ACSL4 and LPCAT3 aid in activating and embedding PUFA into membrane lipids, resulting in PUFA-PL. With the synergistic effect of Fe^2+^, PUFA-PL is oxidized by ROS to produce PUFA-PL-OOH. Research emphasizes the central role of LOX in the oxidation of PUFA-PL. Inhibitors targeting LOX can effectively counteract ferroptosis induced by agents like erastin or RSL3 (17). Ultimately, PUFA-PL-OOH causes damage to the cell membrane, leading to cell death.

### 2.2 Ferroptosis is inhibited in lung cancer

Ferroptosis, although capable of inducing tumor cell death, is prominently suppressed in lung cancer. Studies have identified that in the regulation of the Xc- system, SLC7A11 is overexpressed on the cytoplasmic membrane of NSCLC (18). When SLC7A11 is overexpressed in normal airway epithelial cells, there is an increased glutamine dependence, reduced ROS production, and subsequent ferroptosis suppression, which correlates with a predicted decline in the 5-year survival rate for NSCLC patients (18). In a separate study, it was discovered that LncRNA T-UCR Uc.339 exhibited increased expression levels in patients with LUAD. This upregulation of Uc.339 was found to inhibit ferroptosis and promote LUAD metastasis (19). The mechanism behind this effect involved the inhibition of miR-339, which in turn led to the suppression of the negative regulation of SLC7A11 by miR-339 (19). Zhang Wenjing et al. found that lung cancer tissues exhibit increased expression of RNA binding motif single stranded interacting protein 1 (RBMS1), which may contribute to the evasion of ferroptosis and consequently accelerate the progression of lung cancer (20). Similarly, Lai Yuanyang’s team observed elevated expression of Serine/Threonine/Tyrosine Kinase 1 in NSCLC tissues, and it leads to higher levels of GPX4, promoting cell proliferation and inhibiting ferroptosis (21).

Iron metabolism plays a crucial role in ferroptosis. In both lung cancer tissues and associated cell lines, the expression of iron-sulfur cluster assembly enzyme 1 is enhanced, leading to decreased iron release from the cells and thus inhibition of ferroptosis (22). Serum ferritin (SF) level is increased in NSCLC patients. Lung cancer cells can up-regulate iron-sulfur, inhibit Iron Regulatory Proteins and promote SF expression through NFS1 high expression. Further investigation of this pathway may lead to a better understanding of the mechanism of ferroptosis inhibition in lung cancer.

Lung cancer might also further inhibit ferroptosis by suppressing lipid synthesis. Specifically, lymphoid-specific helicase (LSH) can activate genes associated with lipid metabolism, ferroptosis-related genes and fatty acid desaturase 2, thereby regulating lipid metabolism to inhibit ferroptosis (23). Additionally, a related study found that LSH overexpression decreases intracellular ROS and Fe^2+^ levels, further inhibiting ferroptosis (24).

Despite these insights, research on ferroptosis inhibition in lung cancer remains in its nascent stages, with most studies predominantly focusing on LUAD. Delving deeper into the inhibitory mechanisms across various lung cancer cell lines will undoubtedly enrich our comprehension of lung cancer progression and unveil novel therapeutic strategies.

## 3 Regulation of ferroptosis at different levels in lung cancer

**Figure 2:**
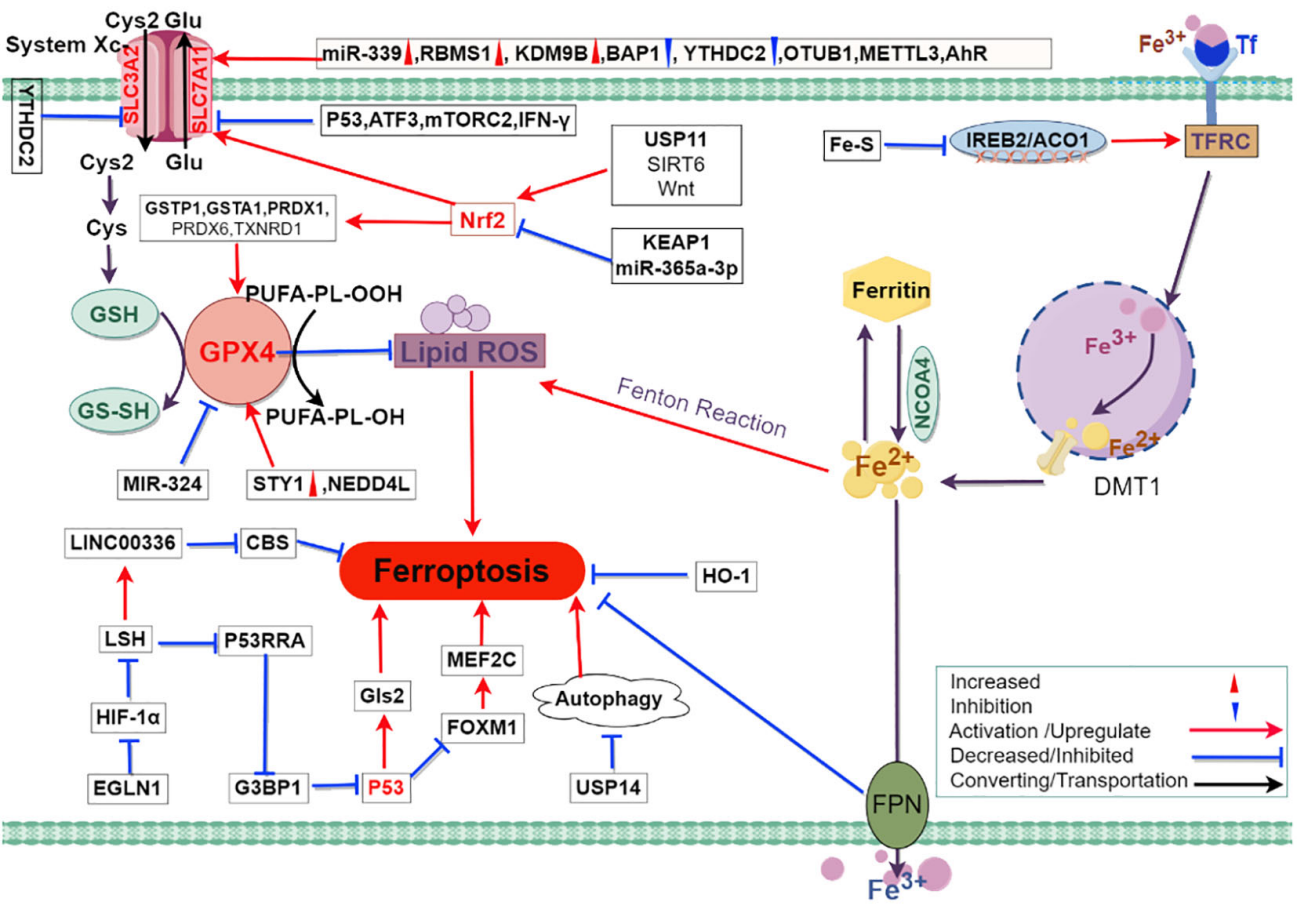
The mechanism of ferroptosis in lung cancer and possible related regulatory pathways. The mechanism of ferroptosis primarily involves iron metabolism, System Xc-, and GPX4. Intracellular iron overload leads to the production of lipid peroxides through the Fenton reaction. System Xc- and GPX4 play a protective role in preventing cell membrane damage caused by lipid peroxides. In the figure, the red triangle marks indicate up-regulated expression of molecules in lung cancer cells, while the blue inverted triangle marks indicate down-regulated expression of molecules in lung cancer cells. The legend in the lower right corner of the figure explains the representation of intermolecular interaction relationships using different lines. Abbreviations: Cys2, cystine; Glu, glutamate; Cys, cysteine; GSH, glutathione; GS-SH, glutathione persulfide; GPX4, glutathione peroxidase 4; SLC3A2, solute carrier family 3 member 2; SLC7A11, solute carrier family 7 member 11; YTHDC2, YTH domain containing 2; PUFAs-OH, hydroxylated polyunsaturated fatty acids; PUFAs-OOH, lipid hydroperoxides; miR-339, microRNA 339; RBMS1, RNA binding motif single stranded interacting protein 1; KDM9B, lysine demethylase 9B; BAP1, BRCA1 associated protein 1; OTUB1, OTU deubiquitinase B1; METTL3, methyltransferase like 3; AhR, aryl hydrocarbon receptor; P53, tumor protein p53; ATF3, activating transcription factor 3; mTORC2, mechanistic target of rapamycin complex 2; IFN-γ, interferon gamma; GSTP1, glutathione S-transferase pi 1; GSTA1, glutathione S-transferase alpha 1; PRDX1, peroxiredoxin 1; PRDX6, peroxiredoxin 6; TXNRD1, thioredoxin reductase 1; USP11, ubiquitin specific peptidase 11; SIRT6, sirtuin 6; Wnt, wingless/integrated; KEAP1, kelch like ECH associated protein 1; miR-365a-3p, microRNA 365a-3p; Nrf2, nuclear factor erythroid 2 related factor 2; ROS, reactive oxygen species; MIR-324, microRNA 324; STY1, stress activated protein kinase STY1/Spc1/Phh1 (fission yeast); NEDD4L, neural precursor cell expressed developmentally downregulated 4 like E3 ubiquitin protein ligase; Fe-S, iron-sulfur; IREB2, iron responsive element binding protein 2; ACO1, aconitase 1; TFR, transferrin receptor; DMT1, divalent metal transporter 1; LINC00336, long intergenic non-protein coding RNA 336; LSH, lymphoid specific helicase; HIF-1α, hypoxia inducible factor 1 alpha; EGLN1, Egl-9 Family Hypoxia Inducible Factor 1; G3BP1, GTPase activating protein binding protein 1; P53RRA, p53 responsive RNA; CBS, cystathionine beta synthase; Gls2, glutaminase 2; FOXM1, forkhead box M1; USP14, ubiquitin specific peptidase 14; HO-1, heme oxygenase 1; FPN, ferroportin.

Ferroptosis is driven by intracellular iron overload through the Fenton reaction, which catalyzes ROS production and induces lipid peroxidation. Ferroptosis in lung cancer cells undergoes multiple aspects of epigenetic, transcriptional, translational, and post-translational modifications (**Figure 2**).

### 3.1 Epigenetic regulation of ferroptosis in lung cancer

The epigenetic control of ferroptosis in cancer is intricate, orchestrated by a combination of DNA methylation, RNA modification, and histone modification. At the forefront of DNA methylation, the LSH emerges as a crucial modulator. Studies illustrate that Egl-9 Family Hypoxia Inducible Factor 1 and c-Myc amplify LSH expression by directly inhibiting HIF-1α (23). LSH has the ability to inhibit ferroptosis by down-regulating the tumor suppressor gene LncRNA-P53RRA in NCSC, which is achieved through the mechanism of LSH interfering with the interaction between P53RRA and GTPase-activating protein-binding protein 1(G3BP1) (25). As a result, TP53 is prevented from being released from the G3BP1 complex, thereby inhibiting the promotion of ferroptosis by TP53 (25).

Beyond DNA methylation, m6A methylation stands as a major RNA modification, pivotal in cancer onset, progression, and metastasis. Specifically, the METTL16-mediated m6A modification drive breast cancer progression by inhibiting ferroptosis through the amplification of GPX4 expression. It has been found that that METTL7B is overexpressed in LUAD cells and can up-regulate GPX4 protein level and enzyme activity through m6A modification, which promotes TKI resistance in NSCLC (26). In addition, Ma et al. found that the m6A Reader YTHDC2 can induce ferroptosis by regulating the expression of SLC7A11 (27).Their also revealed that YTHDC2’s influence on the system Xc- extends further, indirectly suppressing the expression of SLC3A2 via the inhibition of Homeo box A13 (27). The N6-methyladenosine (m6A) modification steered by methyltransferase-like 3 was found to stabilize SLC7A11 mRNA, bolstering its translation. This action not only augments LUAD cell growth but also curtails their ferroptosis, offering a promising target for LUAD diagnosis and therapy (28). Bromodomain-containing protein 4 (BRD4) is capable of detecting acetylated histones, thus facilitating gene transcription by recruiting transcription factors. It is noteworthy that BRD4 inhibitors such as JQ1 and JQ4 have demonstrated the ability to induce ferroptosis in lung cancer cells by reducing the expression of SLC7A11 (29).

In summary, further study of the expression of m6A methylation-related molecules in lung cancer cells and the mechanism of ferroptosis regulation by m6A methylation-related molecules such as SLC7A11 and GPX4 may provide a new feasible pathway for the regulation of ferroptosis in lung cancer.

### 3.2 Transcriptional regulation of ferroptosis in lung cancer

Transcription factors are integral in modulating ferroptosis. One or more such factors can jointly regulate genes associated with ferroptosis, thereby affecting the level of ferroptosis in lung cancer. The current research focus, transcription factor TP53, plays a dual role in regulating ferroptosis. On one front, TP53 suppresses SLC7A11 expression, reducing cystine uptake, promoting glutaminase 2 and heightening cellular sensitivity to ferroptosis. On the other hand, TP53 delayed ferroptosis by upregulating its transcriptional target Cyclin-dependent kinase inhibitor 1A, which inhibited cell cycle progression and slowly consumed glutathione (30). Furthermore, TP53 mutations have been associated with increased resistance to ferroptosis in lung cancer, and this resistance is attributed to the inhibition of forkhead box M1 relieved, which in turn activates myocyte-specific enhancer factor 2C providing stress protection against ferroptosis inducers (31).

Another significant player is Nrf2 that renowned for its antioxidative properties, and its heightened expression shields cancer cells from ferroptosis (32). Inhibition of Nrf2 or promotion of its degradation may be beneficial to ferroptosis induction in lung cancer. For example, it has been found that E3 ubiquitin ligase is overexpressed in some lung squamous cell carcinoma and adenocarcinoma samples, and plays a positive regulatory role in ferroptosis by targeted degradation of NRF2 (33). What’s more, study has shown that NRF2 negatively regulates the expression of focal adhesion protein FOCAD and attenuates its effect on enhancing the sensitivity of NSCLC cells to cysteine deprivation induced ferroptosis, while NRF2 inhibitor Brusatol enhances the therapeutic effect of FOCAD on NSCLC cells (34). Ferroptosis suppressor protein 1 (FSP1) is a transcriptional target of Nrf2. Kelch-like ECH associated protein 1(KEAP1) binds to NRF2 and maintains it at a low level. KEAP1 mutant lung cancer lost its inhibitory effect on NRF2, and when NRF2 is activated, it promotes the expression of FSP1, which inhibits ferroptosis by promoting the reduction of CoQ to CoQH2 (35). It provides a potential therapeutic target for KEAP1 mutant lung cancer. Furthermore, studies have shown that Nrf2 can also regulate the light and heavy chains of ferritin, an iron storage protein (36), and control heme degradation and intracellular iron metabolism through heme oxygenase 1 (HO-1). This suggests that Nrf2 may regulate ferroptosis by influencing iron metabolism, but there is no evidence to support this in lung cancer.

Yes-associated protein serves as a transcriptional co-activator and is suggested to function as an oncogene in LUAD. Through XC- system inhibition, researchers decreased endogenous glutamate build-up, further restrained Yes-associated protein, and assessed LUAD cell sensitivity to ferroptosis, suggesting ferroptosis-based treatments are suitable for late-stage or treatment-resistant LUAD patients (37). Activating transcription factor 3 (ATF3) curtails SLC7A11 expression in a p7-independent manner, accentuating erastin-induced ferroptosis (38). Moreover, the signal transducer and transcription activator 1 activated the transcription of SLC7A11, resulting in an increased expression level of SLC7A11, and epigallocatechin gallate inhibited leppin-induced lung cancer cell growth by down-regulating transducer and transcription activator 1 (39). Latest research suggests the aryl hydrocarbon receptor binding to the SLC7A11 promoter boosts NSCLC progression via SLC7A11 expression activation, augmented cell oxidative sensitivity, and ferroptosis inhibition (40). To conclude, the transcriptional regulation of ferroptosis in lung cancer has unraveled various potential therapeutic targets, furthering the prospects for advanced treatments.

### 3.3 Post-transcriptional regulation of ferroptosis in lung cancer

Long non-coding RNA (lncRNA) and microRNA (miRNA) are critical regulators in the ferroptosis mechanism of lung cancer. Competing endogenous RNA is a post-transcriptional regulation mode. As mentioned above, LncRNA Uc.339 reduces the level of miR-339 by competing with pri-miR-339, alleviating the inhibitory effect on SLC7A11, and promoting the metastasis of lung cancer cells (19). Additionally, contrary to the competing endogenous RNA mechanism, the Metallothionein pseudogene 1 and NrF2-3 ‘-UTR share a consensus binding site on miR-365a-3p, and Metallothionein pseudogene 1 could enhance the inhibitory effect of miR-365a-3p on NRF2 by directly binding to stabilize the RNA inhibition of Mir-365a-3p, thereby promoting ferroptosis in NSCLC (32). It has been reported that ELAVL1 modified LINC00336 through pseudouridine (Ψ) catalyzed by the RNA modification enzyme PUS10 to enhance its expression and inhibit ferroptosis in lung cancer (24). In addition, the sponging effect of miRNA molecules is essential for the post-transcriptional regulation of genes, and circSCN8A reduces its level by sponging miR-1290 and promotes ferroptosis, thereby inhibiting NSCLC proliferation and metastasis (41). Regarding RNA splicing, some articles screened splicing factors related to ferroptosis, such as RBM10 and SRSF2, which can regulate the splicing of ferroptosis-related genes such as TP53 (42). However, there is a lack of evidence for the role of RNA splicing in the regulation of ferroptosis in lung cancer, and this part of the study needs to be further explored. To encapsulate, these revelations shed light on the intricate post-transcriptional regulatory frameworks governing ferroptosis in lung cancer. Delving deeper into these mechanisms will undoubtedly unearth novel treatment targets and pave the way for groundbreaking therapeutic strategies.

### 3.4 Translation regulation of ferroptosis in lung cancer

RNA binding proteins can regulate the translation of genes associated with ferroptosis, thereby altering the sensitivity of lung cancer cells to ferroptosis. Research indicates that RBMS1, as a translation regulator for ferroptosis, promotes the translation of SLC7A11 through its T3 region in the 3′-UTR, leading to an escape from ferroptosis and furthering lung cancer progression (20). In contrast, inhibiting RNA binding motif protein 15 enhances ferroptosis in lung cancer cells through the TGF-β/Smad2 pathway (43). This not only reduces their proliferation but also provides a potential therapeutic approach for lung cancer treatment. Another layer of regulation involves iron regulatory proteins that bind to mRNA sequences containing iron-responsive elements, affecting their translation or degradation. For instance, iron regulatory proteins ACO1 and IREB2 bind to transferrin receptor (TFRC) mRNA and enhance its translation, with their activity being influenced by intracellular iron-sulfur (Fe-S) levels (44). When these Fe-S levels are low, increasing the levels of TFRC protein enhances the cell’s ability to uptake iron, subsequently reducing free intracellular iron and preventing iron-dependent cell death (44). Importantly, tribetidine has been found to up-regulate HIF-1α and iron regulatory protein 1, inhibit GPX expression, and induce ferroptosis, thereby inhibiting the growth of lung cancer cells (45). These findings reveal a potential therapeutic strategy for NSCLC.

### 3.5 Post-translational regulation of ferroptosis in lung cancer

The ubiquitin-proteasome system is pivotal in the post-translational regulation of ferroptosis, with GPX4 being a central molecule in this process. Numerous investigations have highlighted the significant impact of modulating the ubiquitination of GPX4 on the process of ferroptosis in lung cancer. For instance, recent research suggests that Timosaponin AIII forms a complex with Heat Shock Protein 90, resulting in the ubiquitination and degradation of GPX4, thereby inducing ferroptosis in NSCLC (46). Additionally, lactate produced during chemotherapy can inhibit ferroptosis by increasing GPX4 levels. NEDD4 like E3 ubiquitin protein ligase (NEDD4L) binds to GPX4, promoting its ubiquitination and degradation. Lactate can enhance mitochondrial ROS production and activate serum-glucocorticoid-regulated kinase 1, which phosphorylates NEDD4L, thereby reducing its impact on GPX4. These findings reveal the potential role of ferroptosis in chemotherapy resistance (47).

Elevated expression of ubiquitin-specific processing protease 11 has been observed in NSCLC patients (SCC subtype), with its deubiquitinating function stabilizing NRF2, rendering it a crucial molecule for cell proliferation and ferroptosis (48). Additionally, the ubiquitin-specific protease 35 can modulate ferroptosis in lung cancer by targeting iron transport protein (49).The ubiquitination regulation extends to SLC7A11 as well. OTU deubiquitinase B1(OTUB1), an atypical deubiquitinase prevalent in numerous human cancers, principally stabilizes SLC7A11 by impeding its ubiquitination and subsequent proteasomal degradation (20, 50). CD44, serving as an adhesive molecule in cancer stem cells, has a variant, CD44v, that ensures the stability of SLC7A11 protein by adjusting interactions between OTUB1 and SLC7A11 (51). Beyond ubiquitination, SLC7A11 also experiences phosphorylation alterations orchestrated by mechanistic target of rapamycin complex 2 (52).

### 3.6 Relationship between autophagy and ferroptosis in lung cancer

Autophagy, being a significant pathway, regulates ferroptosis, with some research positing ferroptosis as a cell death mechanism contingent on autophagy (53). Numerous studies underline that modulating autophagy can markedly affect ferroptosis in lung cancer. For instance, d-Borneol has the ability to heighten sensitivity to cisplatin by fostering autophagy, instigating ferroptosis, and inhibiting epithelial-mesenchymal transition, thereby amplifying its antitumor efficacy (54). Conversely, a study by Vrushank Bhatt et al. unveiled that escalated autophagy deters ferroptosis, subsequently bolstering lung cancer’s fortitude against Trametinib (55). Thus, this illustrates that autophagy exerts both facilitative and inhibitory regulatory impacts on ferroptosis within lung cancer. However, further research is needed to determine the specific conditions.

## 4 Dual role of ferroptosis in lung cancer

Ferroptosis potentially plays a dual role in both the formation and treatment of tumors. While it generally suppresses tumor growth, there are instances where it can facilitate its progression, contingent upon the tumor’s type and stage (56). Initially, ferroptosis may accelerate tumor progression by inciting inflammatory reactions, yet as the disease advances, it helps restrain tumor expansion through inducing cell apoptosis. This dual nature extends to lung cancer, with a nuanced understanding of ferroptosis’s specific impact offering new avenues for treatment strategies.

### 4.1 Ferroptosis to evade immune surveillance and reduce efficacy

Ye, C. et al. elucidated that Fanconi anemia complementation group D2 (FANCD2), acting as a negative regulator of ferroptosis, is found to be overexpressed in LUAD, which implies a diminished response rate to cancer immunotherapy along with a grim prognosis (57). Ferroptosis can promote tumor growth, proliferation, and help it evade immune surveillance through a variety of mechanisms. Particularly in lung cancer studies, ferroptosis appears to have a correlation with immune infiltration in LUSC, where the process of leukocyte transendothelial migration leads to modulation of M2 macrophages, ensuring their proficient infiltration within the immune microenvironment, thereby exerting an immunosuppressive role and propelling LUSC progression (58). A notable inverse relationship is observed between the majority of immune checkpoint proteins and glyceraldehyde-3-phosphate dehydrogenase (GAPDH) expression. Despite the elevated expression of GAPDH in lung cancer, its manifestation is significantly reduced in ferroptosis scenarios, implying that with the uptick in immune checkpoint protein expression during ferroptosis, tumor cells might navigate immune surveillance more adeptly (59). However, the precise regulatory mechanism of GAPDH in ferroptosis remains veiled, hence, meticulous exploration into its specific function and mechanism could unveil new vistas for lung cancer treatment.

The advent of immunotherapy, portraying a novel treatment paradigm for lung cancer, has ushered in substantial advancements in therapeutic outcomes, extending survival for patients. Delving into the mechanisms and targets exploited by lung cancer to utilize ferroptosis for evading immune monitoring could be instrumental in tackling immunotherapy resistance and catalyzing the innovation of new treatment methodologies.

### 4.2 Potential value of ferroptosis in lung cancer treatment

Ferroptosis has the potential to directly eliminate cancer cells. A large number of studies have shown that inducing ferroptosis combined with conventional treatment can enhance the therapeutic effect and provide a new way for the treatment of lung cancer. There are numerous treatment options that combine ferroptosis with chemotherapy, radiotherapy, and immunotherapy. Furthermore, a range of ferroptosis-related biomarkers are now considered valuable assets in predicting the outlook for lung cancer.

#### 4.2.1 Decreasing chemoresistance

Drug resistance perennially poses a formidable hurdle in the realm of cancer treatment, particularly when employing targeted drugs like gefitinib or platinum-based chemotherapies for lung cancer. A growing body of evidence underscores the immense potential of ferroptosis in diminishing drug resistance in lung cancer treatment.

Platinum agents form the cornerstone of lung cancer chemotherapy. Harnessing ferroptosis to tackle cisplatin-resistant tumor cells may unveil a powerful therapeutic strategy. Li, Y. et al. illustrated that erastin and sorafenib can efficaciously induce ferroptosis in cisplatin-resistant NSCLC cells (N5CP cells), and when utilized either singularly or concomitantly with a lower dose of cisplatin, they significantly hinder the proliferation of N5CP cells (60). Presently, the spotlight in studies aimed at alleviating drug resistance via ferroptosis is trained on GPX4. Deng, S.H. et al. identified that the overexpression of miR-324-3p gene could target GPX4 directly, substantially amplifying cisplatin-induced ferroptosis in cisplatin-resistant A549 (A549/DDP) cells and counteracting their cisplatin resistance (61). Han, N. et al. recognized that ferroptosis triggered by the downregulation of GPX4 via Dihydroartemisinin can notably boost the efficacy of Photodynamic therapy (62). In conclusion, reducing platinum resistance in lung cancer by inducing ferroptosis may be a powerful measure to improve the efficacy of chemotherapy in lung cancer.

EGFR-TKI resistance is associated with ferroptosis escape in LUAD, which targeting GPX4 can promote ferroptosis in EGFR-TKI resistant LUAD (63). In tackling resistance to EGFR-TKI in lung cancer, Manoalide has been shown to effectively enhance the sensitivity of lung cancer cells to osimertinib by inhibiting the NRF2-SLC7A11 axis and inducing ferroptosis by down-regulation of ferritin heavy chain 1 through mitochondrial Ca2+ overload (64). This highlights the potential of MA as an EGFR-TKI sensitizer, particularly in lung cancer cells with KRAS mutations and resistance to Osimertinib (64). The synergistic use of gefitinib and betulinol can escalate the sensitivity of EGFR wild-type/KRAS mutant NSCLC cells to gefitinib (65). Therefore, the use of ferroptosis inducers in EGFR-TKI resistant lung cancer may be another breakthrough.

The stride in nanotechnology has elevated nanomedicine to a pivotal role in lung cancer treatment. Research indicates that marrying nanocatalytic sensitizers with Amyloid Precursor Protein/Death Receptor 6 Inhibitor 12 can surmount the resistance of NSCLC to osimertinib via the ferroptosis pathway and may thwart its metastasis (66). Recently, academia unearthed that 2-methoxy-6-acetyl-7-methyljuglone can trigger ferroptosis by targeting NAD(P)H quinone oxidoreductase 1 (NQO1) in drug-resistant NSCLC cells, opening the door to a new NQO1-mediated treatment modality to battle resistance (67).

#### 4.2.2 Increased radiosensitivity

Ionizing radiation (IR) is known to trigger ferroptosis, a mechanism that can be leveraged to sensitize radioresistant tumors to radiation therapy (68). Particularly in lung cancer, the protein RBMS1 has been implicated in radioresistance by modulating the expression of SLC7A11. Recent investigations have unveiled that N-desmethyl imipramine hydrochloride (NTP) can alter RBMS1 expression in IR-resistant A549 lung cancer cells, thereby enhancing their susceptibility to radiation therapy, which opens a new therapeutic avenue for lung cancer treatment (20). On a related note, heme is recognized for its ability to neutralize free radicals, thereby guarding cells against oxidative harm. Yet, intriguingly, studies reveal that in irradiated lung cancer cells, heme augments the initial generation of ROS, which catalyzes lipid peroxidation, eventually driving ferroptosis and elevating the radiosensitivity of lung cancer cells (69).

The ongoing research continually unveils the interlink between lung cancer’s radiotherapy sensitivity and ferroptosis. Koppula, P. et al. demonstrated that the use of compounds, such as 4-chlorobenzoic acid, can enhance the radiation tolerance of KEAP1, which is irregularly induced, to effectively treat lung cancers with KEAP1 mutations (35). As of now, no drugs targeting the CoQ-FSP1 pathway have entered clinical trials, and this may be the next feasible research direction for lung cancer treatment. Additionally, with nanotechnology progressing, the exploration concerning the nexus between nanomedicine, radiotherapy, and ferroptosis has commenced, paving new avenues for lung cancer treatment. A marked characteristic of the tumor microenvironment is hypoxia. Studies underscore that hypoxia-induced angiopoietin-like 4 (ANGPTL4) hinders ferroptosis via at least two pathways (intracellular ANGPTL4 and hypoxic exosomal ANGPTL4), bestowing resistance to radiotherapy in NSCLC (70). Clearly, channeling focus on ferroptosis to enhance radiation therapy outcomes is a promising route, meriting further scrutiny.

#### 4.2.3 Enhancing the effect of immunotherapy

Immunotherapy, widely acclaimed as the third revolution in the field of cancer treatment, holds significant promise, particularly in the effective management of advanced non-small cell lung cancer. The mechanism by which ferroptosis promotes tumor immunotherapy is that ferroptosis inducers or immune cells down-regulate ferroptosis resistance genes and increase the incidence of ferroptosis, thereby increasing the generation of immunogenic cell death, improving the immunogenicity of tumor, and activating immune cell attack. As observed by Wang, Song et al., a depletion of intracellular cystine can heighten the T-cell-driven anti-tumor immunological response (71). It has been elucidated that there may be some association between the expression of SLC7A11 and SLC3A2, CD8+T cell count, IFN-γ expression and the prognosis of lung cancer patients (72). Upon activation through immunotherapy, CD8+ T cells can enhance lipid peroxidation specific to tumor cells, which could promote ferroptosis, thereby augmenting the anti-tumor efficacy of the immunotherapeutic approach (73). Furthermore, CD8+ T cells can obstruct the expression of genes SLC3A2 and SLC7A11 through the release of interferon-γ, thereby facilitating the initiation of ferroptosis (74). Ferroptosis can also enhance the effect of tumor immunotherapy by regulating the expression of immune checkpoint molecules on the surface of tumor cells, such as PD-L1 and CTLA-4 (75).

The mechanisms by which ferroptosis enhances tumor cell immune escape and promotes tumor immunotherapy are similar in that they both play a role by regulating ferroptosis related genes and acting on immune checkpoints. The difference may be mainly due to the opposite regulatory direction of the two, such as the former upregulates ferroptosis resistance genes and reduces the incidence of ferroptosis, thereby reducing the production of immunogenic cell death, reducing the immunogenicity of tumor, and escaping the killing of immune cells. Recently, it has been reported that Mitochondrial translocator protein promotes tumor cell immune escape by up-regulating PD-L1 expression through Nrf2-mediated transcription (76).

In conclusion, both immunotherapy and ferroptosis manifest substantial potential in the treatment of lung cancer. Persistent, in-depth probing into novel ferroptosis-based immunotherapies and devising new strategies via combination drug therapies may yield significant advantages for the medical community moving forward.

#### 4.2.4 Predicting prognosis

The search for ferroptosis has led to the search for biomarkers to confirm the occurrence and better induction or regulation of ferroptosis. It could refine the stratification of prognosis groups and formulate strategies to counter resistance in radiotherapy, chemotherapy, or immunotherapy. The latest research shows that Jin Ye’s team made a breakthrough and identified the first specific marker of ferroptosis, peroxiredoxin 3 (77). GPX4, SLC7A11, and FSP1 are considered key targets for modulating ferroptosis and could serve as candidate biomarkers for it. Certain studies posit that dysregulation of GPX4, SLC7A11, and Apoptosis-Inducing Factor Mitochondria-Associated 2 occurs in diverse cancers, suggesting their role as prognostic biomarkers for various malignancies. These markers might aid in assessing the immune cell infiltration within tumor tissues. Analytical data coupled with experimental verification underscores a connection between ferroptosis regulatory elements and tumor immune infiltration in LUAD patients, hinting at their potential as promising biomarkers and treatment targets (78). A summary by Peyman Tabnak et al. outlines ferroptosis-related biomarkers in lung cancer, where markers like ACSL3, FANCD2, and SLC7A11 are associated with adverse prognosis and abbreviated overall survival (79).

Recent findings have unveiled new ferroptosis biomarkers in lung cancer. Deng, B. et al. disclosed that Ribonucleotide Reductase Subunit M2 (RRM2) plays a part in ferroptosis, and is upregulated in LUAD cell lines, suggesting its prospective utility as both a biomarker and therapeutic target for immune infiltration in LUAD (80). Thioredoxin-Interacting Protein shows a positive correlation with immune cell infiltration in SCLC, possibly serving as a biomarker for prognosticating treatment outcomes of chemotherapy and immunotherapy in SCLC patients. Li Lei and his team devised a Ferroptosis-Related Gene Pair Index, which may act as an independent prognostic biomarker for personalized tumor treatment (81). FANCD2, acting as a negative modulator of ferroptosis, can be an independent biomarker indicating unfavorable outcomes in LUAD patients (57). Recent research identifies GAPDH as a ferroptosis marker in LUAD cell lines. The amalgamation of immunotherapy with GAPDH-targeted ferroptosis induction in LUAD could unveil a novel therapeutic avenue (59). Furthermore, Hyper-methylated In Cancer 1 (HIC1) in cancers can not only predict the prognosis (including lung cancer) but also predict the effects of immunotherapy and drug sensitivity, marking HIC1 as a potential biomarker with immune activity (82).

Searching for reliable ferroptosis biomarkers in lung cancer holds immense value for predicting lung cancer prognosis. This endeavor not only aids in anticipating the effectiveness of present treatment modalities but also unveils strategies for combined treatments to tackle drug resistance, thus fostering the advancement of new lung cancer treatments. Nevertheless, the implementation of ferroptosis biomarkers in lung cancer encounters numerous hurdles. A majority of these biomarkers are in the screening phase, with a scant few validated for actual clinical application. The biological mechanisms and functions of certain biomarkers, such as GAPDH, demand further scrutiny, and the precise mechanism of action of HIC1 also calls for thorough investigation.

## 5 Ferroptosis-related lung cancer therapies

### 5.1 Medications aimed at lung cancer ferroptosis

Presently, a myriad of drug candidates targeting ferroptosis for lung cancer treatment have been reported. These pharmaceutical agents can be categorized as follows: system Xc- inhibitors that act directly, GPX4 inhibitors, drugs that amplify the Fenton reaction, and those that promote lipid peroxidation. Additionally, certain drugs function indirectly or through a combination of mechanisms. **Table 1** summarizes well-characterized drugs in current studies, offering a consolidated view of their mechanisms and efficacy.

**Table 1:** Drugs targeting ferroptosis in lung cancer.

Type	Drug	Development Phase	Targets	Mechanism/Effect	Reference
System Xc- Inhibitors	Vorinostat	*In vivo*	↓SLC7A11	Promotion of ferroptosis through SLC7A11 downregulation.	(83)
	Dihydroartemisinin	*In vitro* and vivo	↓SLC7A11	Inhibition of the PRIM2/SLC7A11 axis.	(84)
	Artesunate	*In vivo*	↓SLC7A11	SLC7A11 was down-regulated and TFRC was up-regulated.	(85)
	Sulforaphane	*In vitro* and vivo	↓SLC7A11	Inhibition of SLC7A11 mRNA and protein expression levels.	(86)
	Erastin	*In vitro* and vivo	↓SLC7A11	Activation of p53 inhibits SLC7A11 and induces ROS accumulation.	(87)
GPX4 Inhibitors	Cisplatin	*In vivo*	↓GSH, ↓GPX4	Promotes GSH depletion and GPX4 inactivation.	(88)
	Rsl3	*In vitro* and vivo	↓GPX4	Inhibition of GPX4 activity.	(89)
	Sanguinarine	*In vitro* and vivo	↓GPX4	Ubiquitination mediated by E4 ligase STUB4 and degradation of endogenous GPX3 reduced GPX4 stability.	(90)
	Ammonium Ferric Citrate	*In vitro*	↓GPX4	Inhibition of GPX4-GSS/GSR-GGT axis can reduce autophagy and increase Fe^2+^ -induced oxidative stress injury.	(91)
	Dihydroisotanshinone I	*In vitro* and vivo	↓GPX4	Block GPX4 protein expression.	(92)
	Palladium Pyrithione	*In vitro* and vivo	↓GPX4	Increased GPX4 protein degradation.	(93)
	Red ginseng polysaccharide	*In vivo*	↓GPX4	Induces LDH release. Promotes ferroptosis and inhibits GPX4 expression.	(94)
	Auranofin	*In vitro*	↓GPX4	Inhibition of Trx/TrxR system reduced the expression of GPX53 mRNA and GPX273 protein, and up-regulated NrF2-mediated oxidative stress response.	(95)
Induces Fenton reaction and promotes lipid peroxidation	Orlistat	*In vitro* and vivo	↓GPX4, ↑Lipid peroxidation	Decreased GPX4 expression, inhibited FAF2 expression, and induced lipid peroxidation.	(96)
	Zinc	*In vivo*	↑ROS	Induces lipid peroxidation.	(97)
Others	Erianin	*In vitro* and vivo	↑CaM	Activation of Ca^2+^/CaM signaling pathway.	(98)
	Levobupivacaine	*In vitro* and vivo	↑P53	Up-regulation of p53 expression in NSCLC cells induces ferroptosis.	(99)
	Sinapine	*In vivo*	↓SLC7A11, ↑TF/TFR	Upregulation of TF/TFR coupled with downregulation of p53-dependent SLC7A11.	(100)
	Novel quinoline derivative, DFIQ	*In vitro* and vivo	Mitochondria	Increases cellular sensitivity to ferroptosis by impairing autophagic flux, resulting in the accumulation of dysfunctional mitochondria and subsequent induction of ferroptosis.	(101)
	Andrographolide	*In vitro* and vivo	Mitochondria, ↓GPX4 and ↓SLC7A11	Enhanced mitochondrial dysfunction and inhibited GPX4 and SLC7A11 expression.	(102)

“↑”stands for up-regulation or promotion.” ↓” stands for downregulation or inhibition.

SLC7A11, solute carrier family 7 member 11; GPX4, glutathione peroxidase 4; ROS, reactive oxygen species; TFRC, transferrin receptor; PRIM2, primase subunit 2; STUB4, STIP1 homology and U-box containing protein 4; GPX3, glutathione peroxidase 3; GSS, glutathione synthetase; GSR, glutathione reductase; GGT, glutathione transferase; LDH, lactate dehydrogenase Trx, thioredoxin; TrxR, thioredoxin reductase; GPX53, Glutathione peroxidase 53; GPX273, Glycoprotein X273; FAF2, fatty acid synthase 2; CaM, calmodulin; TF, Transferrin; TFR, Transferrin.

As delineated in **Table 1**, a plethora of therapeutic drugs targeting ferroptosis in lung cancer have been reported. However, the lion’s share of these drug candidates remains in the *in vitro* research stage. Monotherapy employing ferroptosis-targeted drugs for lung cancer treatment has not yet been fully realized; hence, combination therapies are often advocated for improved outcomes. Interestingly, synthetic drugs make up a smaller proportion of the therapeutic agents in these studies, while naturally-derived compounds dominate. Looking ahead, the burgeoning field of nanomedicine represents a promising frontier for future research efforts.

### 5.2 Combination therapy strategies aiming at ferroptosis in lung cancer

As previously detailed, enhancing ferroptosis in lung cancer cells substantially elevates their receptivity to diverse treatment protocols. Consequently, an emerging strategy for combating drug-resistant lung cancer involves integrating ferroptosis-targeting drugs with established drug resistance treatments. **Table 2** offers a comprehensive summary of existing reports on such combinatory treatment approaches.

**Table 2:** Co-treatment strategies aiming at ferroptosis in lung cancer.

Drug combination		Clinical Application	Target	Effect	Reference
Cisplatin +	Propofol	No	GPX4	Propofol suppresses ferroptosis mediated by GPX4 via the miR-744-5p/miR-615-3p regulatory axis.	(103)
	Isoorientin	No	SIRT6/Nrf2/GPX4	Isoorientin enhances ferroptosis while overcoming drug resistance in lung cancer via the SIRT6/Nrf2/GPX4 signaling axis.	(104)
	Ginkgetin	No	Nrf2/HO-1, SCL7A11, GPX4	Ginkgo biloba not only intensified ROS production but also deactivated the Nrf2/HO-1 signaling axis, thereby compromising the REDOX homeostasis in cisplatin-treated cells. Additionally, it amplified cisplatin-triggered MMP loss and apoptosis in NSCLC cells.	(105)
	PRLX93936	No	GPX4	Cisplatin combined with PRLX93936 can increase ROS, lipid peroxidation and Fe2+ level, inhibit GPX4 and down-regulate NRF2/Keap1 pathway, and reduce cisplatin resistance.	(106)
Gefitinib +	Dihydroisotansh	No	ROS	Dihydroisotansh treatment resulted in significant upregulation of autophagy, accumulation of ROS, and induction of apoptosis and ferroptosis in a dose-dependent manner.	(107)
	Betulin	No	SCL7A11, GPX4 and FTH1, ROS	Overcoming gefitinib resistance and improving the efficacy of EGFR wild-type/KRAS mutant in NSCLC cells.	(108)
Erastin +	Celastrol	No	ROS, Mitochondria	Co-treatment with low concentrations of erastin and celastrol significantly induced cell death by activating the ROS-mitophagy signaling pathway.	(108)
	Acetaminophen	No	Nrf2/heme oxygenase-1	Regulate Nrf2 nuclear translocation, promote the death of NSCLC cells.	(109)
β-Elemene +	erlotinib	No	ROS, GPX4	Up-regulation of lncRNA H19 induces ferroptosis and enhances the sensitivity of EGFR-TKI resistant lung cancer to erlotinib.	(110)
Auranofin +	Olaparib	No	ROS	Killing of mutant p53 NSCLC cells via lipid peroxidation-dependent ferroptosis.	(111)
Radiotherapy +	Hemin	No	ROS	Increasing the activity of GPX4 degradation enhances the productivity of initial ROS, leading to lipid peroxidation and ferroptosis.	(112)
	Erastin	No	GPX4	Erastin reduces the radiation resistance of NSCLC cells by inhibiting GPX4.	(113)
	Rsl3, imidazole ketone erastin	No	_	Ferroptosis inducers act as radiation sensitizers to enhance the effect of radiation on cytoplasmic lipid peroxidation, leading to cell death.	(114)^*^

^*^ “+” represents the combination.

GPX4, glutathione peroxidase 4; SIRT6, Sirtuin 6; HO-1, Heme oxygenase 1; SLC7A11, solute carrier family 7 member 11; MMP, Matrix metalloproteinase; ROS, reactive oxygen species; KEAP1, kelch like ECH associated protein 1; EGFR, Epidermal growth factor receptor; KRAS, KRAS proto-oncogene; EGFR-TKI, Epidermal growth factor receptor tyrosine kinase inhibitor; FTH1, ferritin heavy chain 1.

Cisplatin is a crucial component of lung cancer chemotherapy. Recent studies have highlighted the effectiveness of combining cisplatin with various ferroptosis inducers. For example, Li, Y. et al. found that low-dose erastin or sorafenib, when used alongside cisplatin, successfully induced ferroptosis in cisplatin-resistant NSCLC N5CP cells (60). Similarly, Liang, Z. et al. observed that the simultaneous use of cisplatin with the erastin analog PRLX93936 suppressed GPX4 expression and triggered ferroptosis (106). Vu, N.T. et al. suggested that the combination of ceramide kinase inhibitors and cisplatin could be a promising therapeutic approach for KRA-mutated NSCLC (115).

Combining ferroptosis-inducing agents with targeted therapies offers another promising avenue. Ishida, T. et al. found that the combined use of the GPX4 inhibitor RSL3 and tyrosine kinase inhibitors might be an effective approach for treating GIST and EGFR-mutated lung cancer (116). Yan, W.Y. et al. suggest that the conjoint utilization of gefitinib and betulinol can transcend the resistance observed in EGFR wild-type/kras-mutated NSCLC cells to gefitinib, heralding a new pathway for the treatment of such lung cancer manifestations (65). Moreover, Xu, C. et al. found that the combination of β-caryophyllene and erlotinib offers a promising treatment strategy for NSCLC patients (110).

Combining the induction of ferroptosis with radiation or immunotherapy holds promise for innovating lung cancer treatment. Integrating immune checkpoint blockade therapies with statin medications has demonstrated a significant enhancement in therapeutic response for NSCLC. It has been observed that when ferroptosis is either inhibited or at a diminished level, tumor cells are inclined to engender an immunologically cold microenvironment (117). Statin medications, known for inducing ferroptosis by restraining the transcriptional expression of PD-L1, mediate an inflammatory tumor microenvironment, thus bolstering anti-PD-1 immunotherapy in NSCLC and unveiling a potential treatment avenue for immune-cold tumors in NSCLC (118).

### 5.3 Nanomedical approaches to treating ferroptosis in lung cancer

The substantial progress in nanomedicine and tumor diagnostic technologies has fostered notable advancements in lung cancer diagnosis and treatment. Employing nanodrugs in lung cancer treatment can amplify the sensitivity of traditional chemotherapy and radiotherapy, while diminishing cancer cell resistance.

Nanomedicine can trigger iron through various mechanisms and the potential of death, such as amplification Fenton reaction, depletion of glutathione, regulating lipid peroxide and combination therapy, and can ensure the smallest drug side effects and customize precise targeting of cancer treatment. Wei, F. et al. conducted a study that focused on the development of CaCO3 nanoparticles loaded with a Pt (IV) prodrug. To improve their water solubility and tumor targeting, the surfaces of the nanoparticles were modified using DSPE-PEG_2000_-Biotin (119). The study investigated various mechanisms involved in the nanoparticle’s action, including mitochondrial Ca2+ overload, glutathione depletion, nuclear DNA platination, ROS and lipid peroxide increment, and highlighted the synergistic effects of apoptosis, ferroptosis, and immunogenic cell death (119). Moreover, Zhu, G. et al. crafted a unique Janus nanoparticle (FTG/L&SMD) that, upon entering tumor cells, employs its glucose oxidase to convert glucose into hydrogen peroxide, which then triggers a reaction with iron to produce hydroxyl radicals, inducing lipid peroxidation and, ultimately, ferroptosis in tumor cells (120). Notably, these nanoparticles exhibited negligible toxicity to normal cells, underscoring their potential for NSCLC treatment. Chen, X. et al. synthesized a chiral ruthenium nanoenzyme (D/L-Arginine@Ru) and used it to enhance macrophage M1 polarization, reversing tumor immune suppression. Additionally, the chiral ruthenium nanoenzyme demonstrated binding with O2 and NO, fighting against tumor activity, and ultimately inducing tumor cell apoptosis and ferroptosis, achieving a “cocktail therapy” for lung cancer (121).

Several methodologies have emerged, proposing the amalgamation of nanomedicines with conventional treatments. For instance, a study by Wang, L. et al. demonstrated that, in conjunction with Amyloid Precursor Protein/Death Receptor 6 Inhibitor 12, the nanocatalytic enhancer (VF/S/A@CaP) significantly curtailed the migration of osimertinib-resistant NSCLC, hinting at a potential nanocatalysis-based methodology for treating severe osimertinib-resistant NSCLC (66). In addition, Wang, J. et al. formulated a metal-organic supramolecular compound (nano PMI@CeO2) capable of restoring TP53 and sensitizing ferroptosis, markedly inhibiting tumor progression in a syngeneic transplant model of lung cancer, while maintaining excellent biocompatibility, thereby presenting a potential candidate drug for cancer treatment (122). Furthermore, the pH-responsive superparamagnetic iron oxide nanoparticle clusters crafted by Li, Y. et al. showcase combined diagnostic and therapeutic capabilities. They fortify on-site iron cell death and apoptosis utilizing radiative treatments and chemodynamic therapy methods (123). Through rational design of nanoparticles and their synergistic combination with various conventional treatments, achieving efficient integrated diagnosis and therapy will become a promising direction for lung cancer treatment.

In conclusion, nanomedicine’s ability to enhance ferroptosis, synergize with standard therapies to bolster treatment responsiveness, mitigate medication resistance, alongside its hallmark of precise treatment approaches and reduced toxicity, harbors immense potential for the diagnosis and treatment of lung cancer.

## 6 Conclusions and discussions

In recent years, the significance of ferroptosis in tumor biology has increased significantly. Ferroptosis is considered to be an emerging field of lung cancer research, providing new perspectives for the occurrence, development and treatment of lung cancer. This article reviews the multifaceted relationship between lung cancer and ferroptosis, and discusses the regulation and potential therapeutic interventions at different molecular levels.

Ferroptosis may be suppressed in lung cancer due to the self-protection of cancer cells. The current research framework of ferroptosis regulation in lung cancer is relatively complete, but there are still some deficiencies in the details. Epigenetic modifications mainly focus on SLC7A11, while the regulation of other factors such as GPX4 still needs further exploration. P53 has a dual effect on ferroptosis, but there is no clear evidence to support its specific role. Tumor cells can promote immune escape and improve the efficacy of immunotherapy by regulating ferroptosis, and the detailed molecular mechanisms need to be further explored. In addition, we found that the development of ferroptosis-targeted drugs for lung cancer has been a hot topic of research, and many chemical and natural drugs have been discovered. However, as shown in **Table 1**, most of the drugs are only validated in cell and animal models, and many drugs only have theoretical positive therapeutic effects and have not been further developed. The in-depth study of ferroptosis has potential for the diagnosis and treatment of lung cancer, reducing chemotherapy resistance, increasing radiation sensitivity, etc. Many combination therapy strategies have been proposed, but as shown in **Table 2**, most of them are only studied *in vitro*, and theoretically provide direction for lung cancer treatment. Doing basic research in the field of ferroptosis in lung cancer is the cornerstone, and translating it to clinical application is the key, and there is still a long way to go. The application of nanomedicine may be a breakthrough for inducing ferroptosis and promoting lung cancer treatment. Most of the nanomaterials are based on the Fenton reaction, and have advantages such as high efficiency, precision and low toxicity in treatment. Attention to the research of nanomedicine in the precise induction of ferroptosis will open up new possibilities for the treatment of lung cancer.

From the perspective of bioinformatics, screening for specific and sensitive ferroptosis-related biomarkers can help to develop targeted therapy strategies and better predict patient outcomes. Jin Ye et al. found the first specific marker of ferroptosis, peroxiredoxin 3 (77), and recent studies have found that PL-PUFA2s can serve as diagnostic and therapeutic targets for regulating ferroptosis, and may become biomarkers of ferroptosis (124). Although GPX4, SLC7A11, FSP1, ACSL3 and FANCD2 can serve as potential biomarkers in lung cancer, they have some guidance for predicting prognosis, but further screening and identification of lung cancer-specific ferroptosis biomarkers still need to be continued, which is of great significance.

In summary, the role of ferroptosis in lung cancer is a current research hotspot. More in-depth exploration of its regulatory mechanisms in lung cancer, finding more targets and specific biomarkers, and developing targeted drugs are the directions we should consider next. Accelerating the clinical development of ferroptosis-targeted drugs and related combination therapy strategies, accelerating the translation of basic research to clinical practice, and truly benefiting lung cancer patients from the research of ferroptosis are our ultimate goals.

## Author contributions

YL: Writing – original draft. XL: Writing – original draft. JL: Writing – review & editing.

## Glossary

**Table:**

ACSL4	Acyl-CoA synthetase long-chain family member 4
ANGPTL4	angiopoietin-like 4
ATF3	activating transcription factor 3
DMT1	Divalent metal transporter 1
EGFR	Epidermal growth factor receptor
EGFR-TKI	Epidermal growth factor receptor tyrosine kinase inhibitor
FANCD2	Fanconi anemia complementation group D2
FIN56	ferroptosis inducer 56
FSP1	Ferroptosis suppressor protein 1
G3BP1	GTPase-activating protein-binding protein 1
GAPDH	Glyceraldehyde-3-Phosphate Dehydrogenase
GPX4	glutathione peroxidase 4
GSH	glutathione
H2O2	hydrogen peroxide
HIC1	Hyper-methylated In Cancer 1
HO-1	heme oxygenase 1
KEAP1	Kelch-like ECH associated protein 1
LOX	lipoxygenase
LPCAT3	lysophosphatidylcholine acyltransferase 3
LPO	lipid peroxides
LSH	lymphoid-specific helicase
LUAD	lung adenocarcinoma
NEDD4L	NEDD4 like E3 ubiquitin protein ligase
NRF2	NF-E2-related factor 2
NSCLC	Non-small cell lung cancer
OTUB1	OTU deubiquitinase B1
PUAF:	polyunsaturated fatty acid
PUFA-PL	polyunsaturated fatty acid phospholipids
PUFA-PL-OOH	polyunsaturated fatty acid phospholipid hydroperoxides
RBMS1	RNA binding motif single stranded interacting protein 1
ROS	reactive oxygen species
RSL3	RAS selective lethal 3
SLC3A2	solute carrier family 3 member 2
SLC7A11	solute carrier family 7 member 11
system Xc-	the cystine/glutamate antiporter system
TFRC	transferrin receptor
TFRC	transferrin receptor
TP53	tumor protein 53
